# Metabolic Analysis of Root, Stem, and Leaf of *Scutellaria baicalensis* Plantlets Treated with Different LED Lights

**DOI:** 10.3390/plants10050940

**Published:** 2021-05-08

**Authors:** Hyeon-Ji Yeo, Chang-Ha Park, Soo-Yun Park, Sun-Ok Chung, Jae-Kwang Kim, Sang-Un Park

**Affiliations:** 1 Department of Crop Science, Chungnam National University, 99 Daehak-Ro, Yuseong-gu, Daejeon 34134, Korea; guswl7627@gmail.com (H.-J.Y.); parkch804@gmail.com (C.-H.P.); 2 National Institute of Agricultural Sciences, Rural Development Administration, Wanju-gun, Jeonbuk 55365, Korea; psy22@korea.kr; 3 Department of Agricultural Machinery Engineering, Graduate School, Chungnam National University, 99 Daehak-Ro, Yuseong-gu, Daejeon 34134, Korea; sochung@cnu.ac.kr; 4 Department of Smart Agriculture Systems, Chungnam National University, 99 Daehak-Ro, Yuseong-gu, Daejeon 34134, Korea; 5 Division of Life Sciences, College of Life Sciences and Bioengineering, Incheon National University, Yeonsugu, Incheon 22012, Korea

**Keywords:** LED lights, medicinal plant, *Scutellaria baicalensis*, flavones, metabolites

## Abstract

Light emitting diodes (LEDs) have recently been considered an efficient artificial light source in plant factories for enhancing plant growth and nutritional quality. Accordingly, this study aimed to review blue, red, and white LED light sources for efficiency and length of the growing period to produce seedlings of *Scutellaria baicalensis* with high nutritional value. The roots, stems, and leaves of *S. baicalensis* seedlings were grown under different LED lights and harvested after two and four weeks, and analyzed using high-performance liquid chromatography and gas chromatography time-of-flight mass spectrometry to identify and quantify primary and secondary metabolites. Roots, particularly in the seedlings treated with white LEDs were determined to contain the greatest concentrations of the representative compounds present in *S. baicalensis*: baicalin, baicalein, and wogonin, which show highly strong biological properties compared to the other plant organs. A total of 50 metabolites (amino acids, sugars, sugar alcohols, organic acids, phenolic acids, and amines) were detected in the roots, stems, and leaves of *S. baicalensis* seedlings, and the concentrations of primary and secondary metabolites were generally decreased with the increasing duration of LED illumination. Therefore, this study suggests that white LED light and a 2-week growing period are the most efficient conditions for the production of baicalin, baicalein, and wogonin.

## 1. Introduction

*Scutellaria baicalensis* Georgi, known as Huang Qin in Chinese medicine, has been used as a conventional herbal remedy in East Asia and is formally listed in the Chinese Pharmacopeia [1]. According to previous research, the root extract of *S. baicalensis* causes apoptosis of hepatocellular, prostatic, pancreatic, urothelial carcinoma, and breast cells, and suppresses the growth of cancer cells in vitro, and it is often used in conjunction with other medicinal plants [2].

Flavonoids are found in vegetables, seeds, nuts, flowers and stems, wine, tea [3], honey, and propolis [4], and the roots of *S. baicalensis* contain flavonoids such as baicalin, baicalein, wogonoside, and wogonin [5]. Baicalin is biosynthesized using several enzymes, including phenylalanine ammonia-lyase (PAL), cinnamate 4-hydroxylase (C4H), 4-coumarate: CoA ligase (4CL), chalcone synthase (CHS), and chalcone isomerase (CHI). It is catalyzed to baicalein through β-glucuronidase (GUS) or vice versa with UDPglucuronate: baicalein 7-O-glucuronosyltransferase (UBGAT) [6]. Similarly, baicalein has in vitro antioxidative, anti-inflammatory, lipoxygenase inhibitory, antiviral, and anti-allergic activities [7]. Wogonin, one of the main chemical components of *S. baicalensis*, is a flavanone derivative containing the nucleus of a phenylbenzopyrone [8] that suppresses tumor growth and angiogenesis in vitro [9].

Artificial light has been known to improve plant development, growth, and phytochemical production; in plant factories that require strong light to grow vegetables, light-emitting diodes (LEDs) are a promising source due to their durability, cool temperature, long life, diverse wavelengths, and small diode size [10]. According to previous studies, LEDs have positive effects on the accumulation of various secondary metabolites, such as glucosinolate, phenylpropanoid, and carotenoid, in *Brassica juncea* sprouts, wheat sprouts, and the callus of *Scutellaria baicalensis* [11,12,13].

However, there are no studies on the effects of LED lights and their duration on metabolites in *S. baicalensis* sprouts. Therefore, this study aimed to investigate the effects of different LED light sources (white, blue, and red) and their duration on metabolic changes in *S. baicalensis* sprouts and to optimize the most efficient qualities for the production of flavones (baicalin, baicalein, and wogonin).

## 2. Results

### 2.1. HPLC Analysis of Baicalin, Baicalein, and Wogonin in Root, Stem, and Leaf of S. baicalensis Treated with Different LED Light Sources

**Figure 1:**
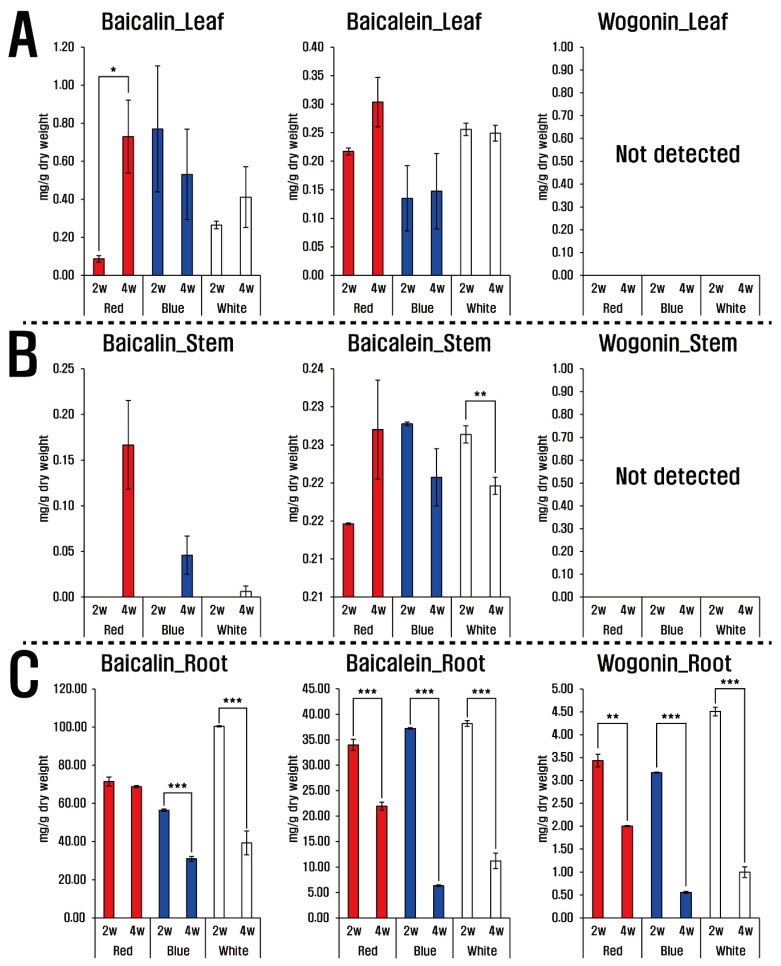
Flavone contents of the leaf (**A**), stem (**B**), and root (**C**) of *S. baicalensis* seedlings grown under LED treatment of varying duration. 2 w and 4 w indicate 2 weeks and 4 weeks, respectively (*t*-test, * *p* < 0.05, ** *p* < 0.01, *** *p* < 0.005).

The three LED lights (red, blue, and white) and their treatment duration caused variations in flavones (baicalin, baicalein, and wogonin) in the roots, leaves, and stems of *S. baicalensis*. Baicalin and baicalein were detected in all plant parts, whereas wogonin was only found in the roots (Figure 1). Roots showed the greatest concentrations of the flavones compared with leaves and stems, and the most abundant was baicalin, followed by baicalein and wogonin. After two weeks under white LED light treatment the roots of *S. baicalensis* seedlings produced the highest levels of baicalin (100.42 ± 0.32 mg/g dry weight (dw)) and wogonin (4.51 ± 0.09 mg/g dw); whereas levels of these compounds decreased in the roots under all three LED colors after four weeks. Similarly, roots under white and blue LED lights, contained slightly higher levels of baicalein than those under red LED light. In stems, baicalin began accumulating after four weeks regardless of light color and those treated with red LED light contained the greatest amounts of baicalin (0.17 ± 0.05 mg/g dw). In contrast, baicalein concentrations showed a slightly increasing accumulation pattern under red LED illumination, whereas stems treated with white and blue LED light revealed decreasing levels with increasing duration. Baicalin and baicalein were also present in leaves, and those treated with red LED light showed increasing patterns of baicalin and baicalein accumulation with increasing illumination duration.

### 2.2. Metabolite-Specific Profiling of Root, Stem, and Leaf of S. baicalensis Treated with Different LED Light Sources

**Figure 2:**
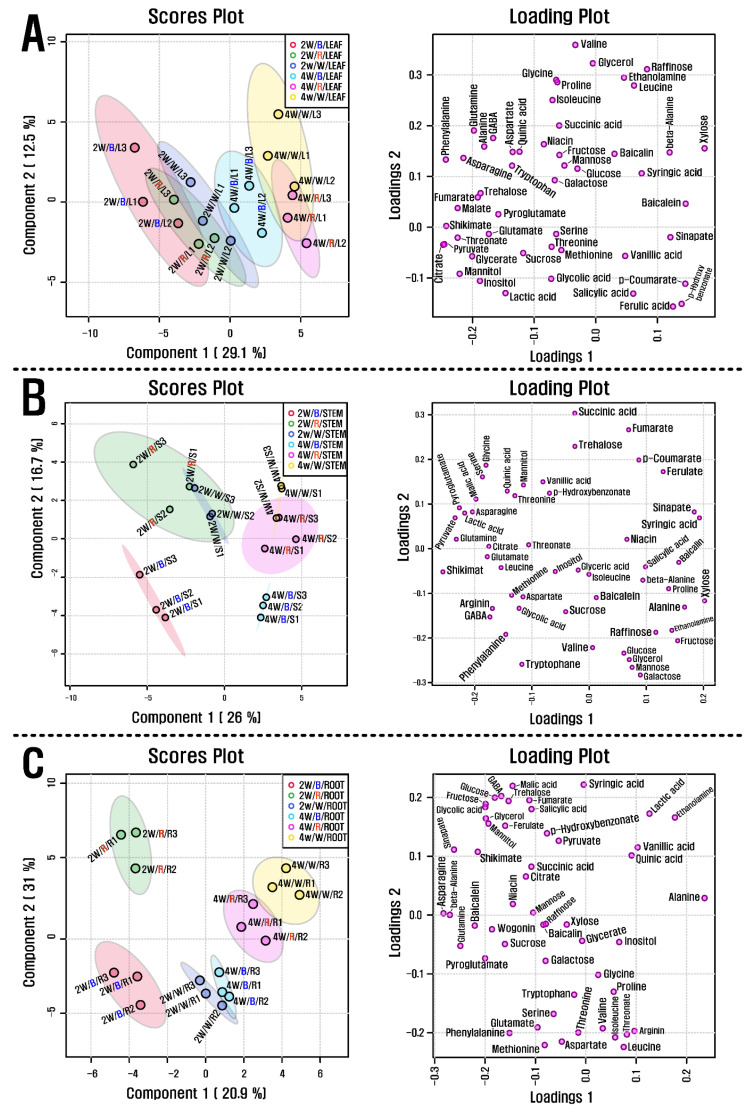
(**A**) Scores and loading plots of the PCA model using metabolites from the leaves of *S. baicalensis* seedlings grown under the LED treatment of varying duration, (**B**) Scores and loading plots of the PCA model using metabolites from the stem of *S. baicalensis* seedlings grown under the LED treatment of varying duration, and (**C**) Scores and loading plots of the PCA model using metabolites from the root of *S. baicalensis* seedlings grown under the LED treatment of varying duration. 2 w and 4 w indicate 2 weeks and 4 weeks, respectively, as well as B, R, and W indicate blue, red, and white, respectively.

GC-TOFMS was used to detect 50 metabolites (amino acids, sugars, sugar alcohols, organic acids, phenolic acids, and amines) in the roots, stems, and leaves of *S. baicalensis* seedlings treated with different LED light sources (red, blue, and white). In leaves and stems treated with blue LED light, a greater number of metabolites were detected than in those treated with red and white LED light. The majority of the amino acids, organic acids, and TCA cycle intermediates showed decreasing patterns in leaves and stems treated with increasing durations of LED light regardless of the source. In contrast, the levels of most sugars and sugar alcohols had slightly increasing patterns in both plant parts. Similarly, roots of seedlings treated with blue or red LED lights contained greater concentrations of metabolites and displayed decreasing patterns of most amino acids, organic acids, and TCA cycle intermediates over time regardless of the light source. White LED light induced slightly increasing levels of sugars and sugar alcohols in the roots, whereas blue and red LED lights revealed decreasing accumulations of these metabolites. Additionally, roots under white LED light for two weeks contained lower levels of sugars and sugar alcohols than those under blue and red LED lights. Partial least-squares discriminant analysis (PLS-DA) was performed with the data derived from GC-TOFMS and HPLC to investigate the metabolic changes in the roots, stems, and leaves of *S. baicalensis* seedlings under various LED light treatments and their durations (Figure 2). The PLS-DA results showed a separation between the leaf group at 2 weeks from that at 4 weeks. This separation was attributable to changes in amino acids, organic acids, sugars, and sugar alcohols as related previously.

**Figure 3:**
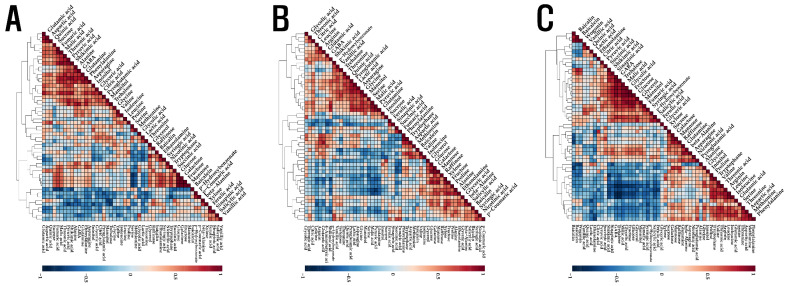
Correlation matrix (**A**) of metabolites obtained from the leaf of *S. baicalensis* seedlings grown under the LED treatment of varying duration, correlation matrix (**B**) of metabolites obtained from the stem of *S. baicalensis* seedlings grown under the LED treatment of varying duration, and correlation matrix (**C**) of metabolites obtained from the root of *S. baicalensis* seedlings grown under the LED treatment of varying duration. Each square indicates the Pearson’s correlation coefficient for a pair of compounds, and the value of the correlation coefficient is represented by the intensity of the deep blue or deep red color, as indicated on the color scale.

To measure the relationship between different metabolites quantified in the roots, stems, and leaves of *S. baicalensis* seedlings treated with different LED lights, an HCA was performed using Pearson’s correlation results (Figure 3). Compounds involved in nitrogen metabolism into amino acids (glutamine, glutamic acid, aspartic acid, and asparagine) and other nitrogen-containing compounds, were positively correlated, and these amino acids and their derivatives were also positively correlated in the roots, stems, and leaves of *S. baicalensis* seedlings treated with different LED lights. Phenylalanine and tryptophan, arising from the shikimate biosynthesis pathway, had a positive relationship with shikimate. The carbohydrates sucrose, galactose, mannose, and raffinose also returned positive correlations. Phenylalanine is a precursor of phenolic acid and flavonoid biosynthesis, and it showed a negative correlation with most phenolic acids and flavonoids. Similarly, most carbohydrates, which act as energy sources, were negatively correlated with most phenolics detected.

## 3. Discussion

In this study, the roots of *S. baicalensis* seedlings treated with white LED light contained the highest levels of baicalin, baicalein, and wogonin and lower levels of most sugars than the other plant parts, suggesting the need for energy to enhance the biosynthesis of phenolic compounds, including the three described here. These results agree with previous studies showing that sugar concentrations for anthocyanin accumulation were lower in purple kohlrabi than in green kohlrabi [14] and that a fungal elicitor allowed for the more rapid depletion of sugar pools to promote alkaloid biosynthesis in cell cultures of *Papaver somniferum* [15].

Numerous previous studies have reported that LED illumination can enhance secondary metabolite production in vegetables and medicinal plants. White LED illumination has been shown to increase the accumulation of phenolics in *Agastache rugosa* seedlings [16], carotenoids in *Fagopyrum tataricum* sprouts [17], and glucosinolates in *Brassica juncea* sprouts [11], compared with other colored LED lights, consistent with the findings of this study. Blue LED light has been reported to increase accumulations of phenolics in *Brassica napus* [18] and *Glycine max* sprouts [19], and red LED light has been shown to enhance both phenolic compounds in the leaves of *Myrtus communis* in vitro [20] and carotenoid production in the outer peel layer of citrus fruit [21].

The metabolic networks of glutamine, glutamate, aspartate, and asparagine are involved in various nitrogen-related processes, including nitrogen assimilation by plants, metabolism into amino acids and other nitrogen-containing compounds, transport between source and sink, stress-associated metabolism, and carbon-nitrogen partitioning. Glutamine is derived from ammonium assimilation and can be converted into glutamic acid with α-ketoglutarate, a TCA cycle intermediate. This glutamic acid is further metabolized into aspartic acid, which is converted to asparagine. The first three compounds can be used for the synthesis of proteinogenic and non-proteinogenic amino acids, amides, and other nitrogenous compounds. Asparagine is a prominent nitrogen transport agent as well as a proteinogenic amino acid [22,23,24].

In this study, glutamate, glutamine, asparagine, and aspartate showed decreasing concentration levels in the roots, leaves, and stems of *S. baicalensis* seedlings treated with different LED lights, with a related reduction of their derivatives. This result was supported by the positive correlations between these four compounds and their derivatives. Furthermore, since shikimate and phenylalanine, which are derived from the shikimate pathway, were negatively correlated with most phenolic compounds, the biosynthesis of these compounds, including phenolic acids and flavones must have been assisted by intermediates or precursors. These findings corroborate a previous study reporting that the internal pool of phenylalanine was lower in purple kohlrabi, which contained a high amount of phenolic compounds, reflecting a precursor supply to produce phenolic acids and anthocyanins [14].

Artificial LED source is important to regulate the lighting systems in a plant factory to produce high-quality plant materials. Therefore, this study suggests that *S. baicalensis* seedlings, containing a high number of health-beneficial compounds, can be produced under LED lights in limited space since *S. baicalensis* was generally cultivated in the field and indicates that the optimal light was white LED for flavone accumulation in *S. baicalensis* seedlings.

## 4. Materials and Methods

### 4.1. Preparation of Plant Materials

*S. baicalensis* seeds were purchased from Aram Seed Co. (Seoul, Korea). Seeds for germination were soaked overnight in water. To produce seedlings, 50 seeds were placed in each pot (diameter: 12 cm, height: 11 cm) containing vermiculite and grown in a growth chamber equipped with fluorescent light with a flux rate of 35 µmol·m^−2^·s^−1^ at 25 °C. After 2 weeks, the seedlings in six pots were moved to a room in a growth chamber equipped with each blue, white, and red LED light with a flux rate of 90 µmol·m^−2^·s^−1^ at 25 °C with an 8 h dark/16 h light cycle. The leaves, stems, and roots from seedlings were harvested with liquid nitrogen and then freeze-dried for further metabolite analysis after two and four weeks of LED light treatment. The LED light sources, and their specific information are described in Appendix A Table A1 and a previous study [18]. Seedlings from three pots were used as independent replicates for each LED light for each duration.

### 4.2. High-Performance Liquid Chromatography (HPLC) Analysis for Flavones

We detected three flavones (baicalin, baicalein, and wogonin) using a slightly modified method of Park et al., [25]. The freeze-dried samples were ground into a powder using a grinder (Wonder blender WB-1, SANPLATEC CORP, Osaka, Japan). The *S. baicalensis* root, stem, and leaf powders (0.1 g each) from seedlings treated with various LED lights were extracted with 2 mL of 80% (*v*/*v*) aqueous MeOH and vortexed for 30 s. Following sonication for 1 h, the samples were centrifuged at 10,000× *g* at 4 °C for 20 min, and the crude extracts were syringe-filtered to a vial for analysis. The HPLC system and analysis conditions were the same as those used in the method reported by Park et al. [25] (Table A2). The three different flavones were identified by retention time and spike tests, and the equation of calibration curves for each flavone was obtained to quantify the compounds in the roots, stems, and leaves of *S. baicalensis* seedlings treated with the different LED lights.

### 4.3. Gas Chromatography Time-of-Flight Mass Spectrometry (GC-TOFMS) Analysis

Hydrophilic metabolites were detected using the method reported by Park et al. [26]. The root, stem, and leaf powders (0.1 g each) of *S. baicalensis* seedlings treated with different LED lights were extracted with 2 mL of 80% (*v*/*v*) aqueous MeOH and vortexed for 30 s. After sonication for 1 h, each sample was centrifuged at 10,000× *g* at 4 °C for 20 min, and then the crude extracts were syringe-filtered into a vial for analysis. The system and analysis conditions were reported by Park et al., 2021. Retention time comparison and spike test were conducted to identify the three different flavones, and the equation of calibration curves for each flavone was obtained to quantify the compounds in the roots, stems, and leaves of the *S. baicalensis* seedlings. The tissue powders (0.01 g each) were placed in a 2 mL tube along with 1 mL of a water/chloroform/methanol mixture (1:1:2.5 *v*/*v*/*v*) and 60 μL of ribitol (0.2 g/L; Sigma, St. Louis, MO, USA) as an internal standard. The extracts were mixed at 1200× *g* using a thermomixer, followed by centrifugation at 10,000× *g* for 5 min. The polar phase (0.8 mL) was transferred to a fresh tube containing water for chromatography (0.4 mL) and evaporated for 3 h. The dried residues were derived by adding 0.08 mL of methoxyamine hydrochloride/pyridine (20 g/L), followed by shaking at 37 °C for 2 h. After the addition of 0.08 mL of *N*-methyl-*N*-(trimethylsilyl)trifluoroacetamide, each tube was heated at 37 °C for 30 min. The final extract was placed in a vial for GC analysis. The analysis system, condition, and program of GC-TOFMS were used to identify and quantify metabolites in the roots, stems, and leaves of *S. baicalensis* seedlings treated with different LED lights according to the previous studies [26,27].

### 4.4. Statistical Analysis

SPSS (version 24.0;(IBM, Chicago, IL, USA)) was used to perform a t-test and MetaboAnalyst 5.0 (http://www.metaboanalyst.ca/, accessed on 5 March 2021) was used for principal component analysis (PCA) and hierarchical cluster analysis (HCA) using Pearson correlations for the metabolites detected in roots, stems, and leaves of *S. baicalensis* seedlings treated with different LED lights. The resolution of the resulting figures was improved using Adobe Illustrator.

## 5. Conclusions

Considering flavone content, white LED light for 2 weeks was the most efficient for the production of the three different flavones in the roots, stems, and leaves of *S. baicalensis* seedlings. Based on the results from the current and previous studies, it appears that the effect of different LED lights on the accumulation of secondary metabolites may depend on plant species, and this study reports that white LED lights are the most optimal for flavone accumulation in *S. baicalensis* seedlings.

## Appendix A

**Table A1:** Plant growth method using LED lights.

LED Plant Growth Chamber	
Product	Multi-Room Chamber HB-302S-4 (Hanbaek Scientific Co.,)
Picture, which is taken from a previous study [10]	
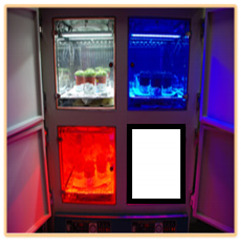
Dimension of each room (L × W × H)	136 cm × 78 cm × 168 cm
LED lights	The white (450–660 nm), blue (450 nm), or red (660 nm) LED lights (PGL-PFL series) were manufactured from PARUS LED Co., Cheonan, Korea

**Table A2:** HPLC analysis method.

HPLC Analysis Performed Using Our Previous Study [25]	
Equipment	NS-4000 HPLC apparatus (Futecs, Daejeon, Korea)
Detector	UV-Vis
Column	optimapak C18 column (250 mm × 4.6 mm, 5 µm; RStech, Daejon, Korea)
Detector wavelength	275 nm
Oven temperature	30 °C
Flow rate	1.0 mL/min
Mobile phase	Acetonitrile, solvent A and 0.2% (*v*/*v*) acetic acid, solvent B
Gradient program	Solvent B 90%; 0 min, solvent B 80%; 10 min, solvent B 80%; 15 min, solvent B 75%; 20 min, solvent B 75%; 25 min, solvent B 40%; 50 min, solvent B 90%; 50.1–60 min
